# Activities of daily living and its influencing factors for older people with type 2 diabetes mellitus in urban communities of Fuzhou, China

**DOI:** 10.3389/fpubh.2022.948533

**Published:** 2022-09-26

**Authors:** Jin-Hua Jie, Dan Li, Li-Na Jia, Yifeng Chen, Yan Yang, Bailing Zheng, Chuancheng Wu, Baoying Liu, Rongxian Xu, Jianjun Xiang, Hai-Lin Zhuang

**Affiliations:** ^1^Department of Health Management, Fujian Health College, Fujian, China; ^2^Department of Preventive Medicine, School of Public Health, Fujian Medical University, Fujian, China; ^3^Department of Nutrition and Food Safety, School of Public Health, Fujian Medical University, Fujian, China

**Keywords:** type 2 diabetes, ADL, elderly, functional limitation, comorbidities, Fuzhou

## Abstract

**Background:**

Type 2 diabetes mellitus (T2DM) is an independent risk factor for functional limitations among the older population. The predicted increase in T2DM cases combined with the ongoing rapidly aging population may further burden the already overloaded healthcare system and aggravate the loss of economic self-sufficiency. This study aimed to investigate the activities of daily living (ADL) and its influencing factors on older people with T2DM, and to provide implications for the development and improvement of community nursing services in the context rapidly aging population in China.

**Methods:**

From March 2019 to June 2020, we conducted a cross-sectional questionnaire survey among older T2DM patients in Fuzhou, using a multi-stage cluster sampling approach. Functional status was measured by the Lawton ADL scale. Stata “nptrend” test was used to examine the trend of ordinal variables on ADL. Non-conditional logistic regression was used to identify factors affecting ADL limitations.

**Results:**

A total of 2016 questionnaires were received, with a response rate of 96%. 12.4% of participants suffered from varying degrees of functional impairment. ADL limitations increased with age. More comorbidities were associated with a greater risk of developing functional limitations in ADLs. the following sub-groups were more likely to suffer from ADL impairment: those aged 70 and over years (OR = 1.99, 95%CI 1.77–2.56), living in an aged care house or with spouse/children (OR = 2.31, 95%CI 1.25–4.26), low monthly income (OR = 1.49, 95%CI 1.28–1.64), without health insurance (OR = 1.82, 95%CI 1.40–2.40), tight family expenses (OR = 1.95, 95%CI 1.42–2.69), having stroke (OR = 6.70, 95%CI 2.22–20.23) or malignant tumor (OR = 4.45, 95%CI 1.27–15.53), irregular eating habit (OR = 2.55, 95%CI 2.23–2.92), smoking (OR = 1.40, 95%CI 1.22–1.60), sedentary lifestyle (OR = 2.04, 95%CI 1.46–2.85), lack of physical exercise (OR = 1.35, 95%CI 1.19–1.53), sleeping difficulty (OR = 1.25, 95%CI 1.10–1.42), and lack of family support (OR = 1.19, 95%CI 1.10–1.29).

**Conclusion:**

Older adults (≥70 years) with T2DM had a high prevalence of functional limitations across a range of daily living tasks, which not only affect individual life of quality but also present a huge burden on the family, health services system, and the whole society. Identified factors associated with ADL limitations may provide useful information for targeted nursing practice and health promotion.

## Introduction

Type 2 diabetes mellitus (T2DM) is a serious public health concern. According to the latest 10th Edition IDF (International Diabetes Federation) Report (1), about 537 million people were living with diabetes (over 90% being T2DM) worldwide in 2021 and ~6.7 million people have died from it or its complications at the same year. Driven by a complex interplay of multifarious factors such as the rapidly aging population, increased sedentary lifestyle, and abrupt changes in traditional dietary habits (2), T2DM has become one of the fastest-growing global health emergencies in this century, with the projected prevalence rate reaching 7,079 individuals per 100,000 by 2030 (3). China has the largest numbers of both current (accounting for about one-quarter of global cases) and projected T2DM cases partly due to its large population size (1). From 1990 to 2016, the all-age morbidity and mortality rates in China have dramatically increased by 78.4 and 63.5% (4), respectively, presenting huge healthcare and economic burden on the society.

T2DM is a prevalent chronic health condition more frequently affecting people aged 65 and over. Results of the 2017 national epidemiological survey showed that the prevalence of diabetes was 30.2% in people aged ≥60 years and the pre-diabetes prevalence rate reached 47.7%, although the proportion of undiagnosed cases was estimated to be 51.7% (1). Older T2DM patients were found to have an accelerated decline in leg lean mass, muscle strength and functional capacity when compared with normoglycemic control groups (5). Evidence has shown that T2DM is an independent risk factor for functional limitations among the older population (6), impairing about 60% of activities of daily living (ADL) for diabetic people aged>65 years compared with only 34% for the same age group without T2DM in the USA (7), especially among older Mexican Americans with T2DM (8). Moreover, T2DM patients were two to three times more likely to suffer from disability than their counterparts (9), and utilized healthcare services more frequently as well. Therefore, the predicted increase in T2DM cases combined with the ongoing rapidly aging population may further burden the already overloaded healthcare system and aggravate the loss of economic self-sufficiency.

Effective diabetes self-management is critical for maintaining health and preventing the occurrence of further diabetes-related complications such as diabetic ketoacidosis, hypoglycemia, cardiovascular diseases, retinopathy, nephropathy, vascular nephropathy, and foot complications (6). However, self-management can be especially challenging for elderly people with T2DM, as they are more likely to suffer from functional limitations and develop geriatric syndromes than those without diabetes (8, 9). Results of a survey including 1,691 individuals sampled from 5 provinces of China showed that T2DM patients especially those living in a low socioeconomic status were moderately satisfied with urban community health services (10), indicating room to improve diabetes caring services at a community level in the aspects of healthcare services quality, health promotion, health insurance, and the essential drug system (10, 11). Currently, few studies in China have investigated the extent to which older adults suffer from functional limitations due to T2DM and its related complications. This study aims to investigate older T2DM patients' activities of daily living (ADL) and identify its influencing factors. Findings of this study can provide useful evidence for the provision of targeted community healthcare services for older people with T2DM to improve their quality of life.

## Materials and methods

### Study design and participant recruitment

Located on the southeast coast of China, Fuzhou is the capital city of Fujian Province, with a population of around 8 million in 2020. In line with the national trend, Fuzhou has stepped into an aging society since 2000 (12). The aging of Fuzhou's population is still ongoing at a rapid pace, and the proportion of people aged >60 years increased from 12.1% in 2011 to 19.1% in 2020 (13). In terms of T2DM, the age-standardized prevalence rate (12.3%) of T2DM in Fuzhou was slightly higher than the national average level (11.2%) in 2017 and similarly for the age-standardized mortality rate (14.7 per 100,000 vs. 12.7 per 100,000) (14–16). Since 2009, Chinese government launched the National Basic Public Health Service Program (NBPHSP) to provide 14 categories of health services to all urban and rural residents free of charge. NBPHSP covers the management of T2DM patients, including screening, regular follow-up, and health education (17). Local community health service centers and village clinics are responsible for the provision of NBPHSP services.

From March 2019 to June 2020, we conducted a cross-sectional questionnaire survey among T2DM patients in Fuzhou to investigate their ADLs. T2DM patients were approached under the support of local community health service centers when they carried out the NBPHSP services for T2DM patients. In this study, T2DM patients were recruited through a multi-stage random cluster sampling process. Firstly, two of the five urban districts in Fuzhou were randomly selected through drawing lots (Names of the five districts were put into a bowl and two names were randomly chosen). The two sampled districts (Taijiang District and Gulou District) have 22 community health service centers; Secondly, we assigned a unique number from 1 to 22 to each of the 22 community health service centers. Then, 11 of the 22 community health service centers were randomly selected as our study sites through an online random number generator (https://epitools.ausvet.com.au/randomnumbers). The inclusion criteria were T2DM patients aged ≥60 years. Those could not answer the survey questions because of health issues (e.g., dementia or/and mental disorders) were excluded. Participation was completely voluntary and no incentives were offered. Informed consent was obtained from individual participants. The study has been approved by the Medical Ethics Committee of Fujian Health College.

### Questionnaire design

The questionnaire consists of two parts. The first section requested the following demographic information: age, gender, education level, marital status, household income, comorbidities, family support, and individual living habits (e.g., smoking, drinking, sleeping, physical exercise, eating pattern). The second section is the widely used Lawton ADL scale (validated Chinese version) to measure two important domains of functioning of older people with T2DM: physical self-maintenance scale (PSMS) and instrumental activities of daily living (IADL) (18). PSMS contains ratings of self-care ability necessary for living in the community in areas of toileting, feeding, dressing, bathing, and locomotion. In contrast, IADL contains a more complex set of behaviors required for independent living skills, including the following eight areas: telephoning, shopping, food preparation, housekeeping, laundering, use of transportation, use of medicine, and financial behavior. Each item of PSMS and IADL was measured using a 4-point Likert scale question: “do it completely by yourself,” “a little difficult to do it independently,” “do it with assistance,” and “must be done by others.” They were assigned 1–4 scores, respectively. Therefore, PSMS has a summary score from 4 to 24 and IADL has a summary score from 8 to 32. The higher the score means the greater the person's functional limitation. ADL consists of PSMS and IADL, with a summary score from 14 to 56. The severity of ADL was classified into three levels: normal (14 scores), somewhat impaired (15–21 scores), and severely impaired (≥22 scores). PSMS and IADL were defined as “impaired” if the summary scores exceeded 6 and 8, respectively.

After a pilot survey, the questionnaire was revised to ensure all questions were clear and understandable. The questionnaire has also been reviewed by relevant experts. All investigators received unified training to ensure the survey was carried out consistently. Participants filled out the questionnaire by themselves under the support/assistance of an on-site investigator in the local community health service center. Completed questionnaires were checked and collected by the investigators on the spot.

### Statistical analysis

Data entry was facilitated using EpiData 3.1 software (EpiData Association, Odense M, Denmark). The demographic characteristics of ADL were descriptively analyzed. The scores of functional impairments were summarized as mean ± standard deviation. Kruskal-Wallis H test and Mann-Whitney *U* test were conducted as the first step to identifying factors associated with ADL. Stata “nptrend” test was used to examine the trend of ordinal variables on ADL (19). Then, we put statistically significant factors into a non-conditional logistic regression model to identify the factors influencing ADL (inclusion criterion α = 0.05, elimination criterion α = 0.10). Stata 16.0 was used to perform all statistical analyses. Results were considered statistically significant at a *P* < 0.05.

## Results

A total of 2,016 questionnaires were received, with a response rate of 96%, including 995 participants recruited from Taijiang District and 1,021 participants from the Gulou District. As shown in Table 1, the average ADL self-care ability score was 14.91 ± 3.38 points. 12.4% of participants suffered from varying degrees of functional impairment, of which 8.2% were mild and 4.2% were severe. The average points for PSMS and IADL were 6.20 ± 1.10 and 8.71 ± 2.49, respectively. Accordingly, the percentages of participants with impaired function for PSMS and IADL were 5.2 and 12.0%, respectively.

**Table 1:** Functional limitation in older people with type 2 diabetes in Fuzhou City.

**Dimension**	**Item**	**Normal**	**Impaired**	**Impaired rate**
		***n* (%)**	***n* (%)**	***n* (%)**
PSMS (scores) 6.20 ± 1.10	Dressing	1976 (98.0)	40 (2.0)	104 (5.2)
	Grooming	1977 (98.1)	39 (1.9)	
	Feeding	1980 (98.2)	36 (1.8)	
	Physical ambulation	1942 (96.3)	74 (3.7)	
	Toilet	1953 (96.9)	63 (3.1)	
	Bathing	1942 (96.3)	74 (3.7)	
IADL (scores) 8.71 ± 2.49	Ability to use telephone	1935 (96.0)	81 (4.0)	242 (12.0)
	Shopping	1869 (92.7)	147 (7.3)	
	Food preparation	1869 (92.7)	147 (7.3)	
	Housekeeping	1839 (91.2)	177 (8.8)	
	Laundry	1838 (91.2)	178 (8.8)	
	Mode of transporation	1899 (94.2)	117 (5.8)	
	Responsibility for own medications	1921 (95.3)	95 (4.7)	
	Ability to handle finances	1907 (94.6)	109 (5.4)	
ADL (scores) 14.91 ± 3.38	1766 (87.6)	250 (12.4)

PSMS refers to “physical self-maintenance scale,” IADL refers to “instrumental activities of daily living,” and ADL refers to “activities of daily living”. The scores of PSMS, IADL, and ADL were summarized as mean ± standard deviation; and ADL, PSMS and IADL were defined as “impaired” if the summary scores exceeded 14, 6, and 8, respectively.

Differences in the functional capability of participants with T2DM by demographic characteristics are summarized in Table 2. We found ADL functions were significantly affected by the following demographic factors: age (*H* = 52.13, *P* < 0.01), gender (*Z* = −2.53, *P* = 0.01), marital status (Z = −4.19, *P* < 0.01), monthly income (*H* = 68.31, *P* < 0.01), and health insurance status (*H* = 14.41, *P* < 0.01). Moreover, with the increase in age ADL functions demonstrated a decreasing trend (*Z* = 12.06, *P* < 0.001). Conversely, education level (Z = −2.4, *P* = 0.017) and financial status (*Z* = −3.99, *P* < 0.001) showed an increasing trend with ADL functions.

**Table 2:** Comparison of activities of daily living (ADL) by demographic characteristics in older people with type 2 diabetes.

**Item**	***n***	**ADL**			***H* /*Z***	***P*-value**
		**Normal**	**Somewhat impaired**	**Severely impaired**		
Age (years)*					52.13	< 0.001
60–69	902	824 (91.4)	67 (7.4)	11 (1.2)		
70–79	835	731 (87.6)	62 (7.4)	42 (5.0)		
≥80	279	211 (75.6)	36 (12.9)	32 (11.5)		
**Gender**					−2.53	0.01
Male	910	779 (85.6)	82 (9.0)	49 (5.4)		
Female	1,106	987 (89.2)	83 (7.5)	36 (3.3)		
**Marital status**					−4.19	< 0.001
Married	1,779	1,578 (88.7)	136 (7.6)	65 (3.7)		
Alone	237	188 (79.3)	29 (12.2)	20 (8.5)		
Education level*					1.19	0.88
Illiteracy	155	134 (86.4)	15 (9.7)	6 (3.9)		
Primary school	422	364 (86.3)	41 (9.7)	17 (4.0)		
Junior high school	648	569 (87.8)	50 (7.7)	29 (4.5)		
Senior high/technical school	550	486 (88.4)	40 (7.3)	24 (4.3)		
College and above	241	213 (88.4)	19 (7.9)	9 (3.7)		
Monthly income (Yuan)*					68.31	< 0.001
< 1,000	207	163 (78.7)	24 (11.6)	20 (9.7)		
1,000–1,999	244	181 (74.2)	51 (20.9)	12 (4.9)		
≥2,000	1,565	1,422 (90.9)	90 (5.7)	53 (3.4)		
Health insurance*					14.41	< 0.001
Completely covered	256	240 (93.8)	10 (3.9)	6 (2.3)		
Partially covered	1,724	1,499 (86.9)	148 (8.6)	77 (4.5)		
Not covered	36	27 (75.0)	7 (19.4)	2 (5.6)		
Total	2,016	1,766 (100.0)	165 (100.0)	85 (100.0)		

^*^Refers to Kruskal-Wallis H test; otherwise, it is Mann-Whitney U test; H/Z is the statistical parameter of Kruskal-Wallis H test and Mann-Whitney U test, respectively; The severity of ADL was classified into three levels: normal (14 scores), somewhat impaired (15–21 scores), and severely impaired (≥22 scores).

Table 3 summarizes the differences in ADL functions by individual behaviors. We found the following factors significantly affected ADL functions of older people with T2DM: eating habits (*H* = 138.92, *P* < 0.001), smoking (*H* = 14.88, *P* < 0.001), drinking (*Z* = −3.28, *P* < 0.001), doing exercise (*H* = 32.82, *P* < 0.001), sedentary lifestyle (*Z* = –7.66, *P* < 0.001), and sleeping difficulty (*H* = 29.17, *P* < 0.001). Results of trend analysis show that those eating more regularly (*Z* = 17.77, *P* < 0.001) and doing more exercise (*Z* = –10.76*, P* < 0.001) had relatively fewer ADL function restrictions.

**Table 3:** Comparison of activities of daily living (ADL) functions by individual behaviors in older people with type 2 diabetes in Fuzhou.

**Variable**	***n***	**ADL**			***H* /*Z***	***P*-value**
		**Normal (*n* = 1,766)**	**Somewhat impaired (*n* = 165)**	**Severely impaired (*n* = 85)**		
Eating habit*
Very regular	1,257	1,183 (94.1)	47 (3.7)	27 (2.2)	138.92	< 0.001
Regular	653	511 (78.2)	99 (15.2)	43 (6.6)		
Irregular	106	72 (67.9)	19 (17.9)	15 (14.2)		
Smoking*					14.88	< 0.001
Yes	171	137 (80.1)	26 (15.2)	8 (4.7)		
No	1,742	1,546 (88.8)	126 (7.2)	70 (4.0)		
Ex-smoker	103	83 (80.6)	13 (12.6)	7 (6.8)		
**Drinking**					−3.28	< 0.001
Yes	117	91 (77.8)	18 (15.4)	8 (6.8)		
No	1,899	1,675 (88.2)	147 (7.7)	77 (4.1)		
Doing exercise*					32.82	< 0.001
Frequently	854	779 (91.2)	59 (6.9)	16 (1.9)		
Occasionally	883	768 (87.0)	67 (7.6)	48 (5.4)		
Never	279	219 (78.5)	39 (14.0)	21 (7.5)		
**Sedentary hours per day**					−7.66	< 0.001
< 8	1,503	1,365 (90.8)	100 (6.7)	38 (2.5)		
≥8	513	401 (78.1)	65 (12.7)	47 (9.2)		
Sleeping difficulty*					29.17	< 0.001
Frequently	323	292 (90.4)	18 (5.6)	13 (4.0)		
Occasionally	933	777 (83.3)	108 (11.6)	48 (5.1)		
No	760	697 (91.7)	39 (5.1)	24 (3.2)		
Participate in community activities*				3.44	0.18
Frequently	321	288 (89.7)	29 (9.0)	4 (1.3)		
Occasionally	948	817 (86.2)	95 (10.0)	36 (3.8)		
Never	747	661 (88.5)	41 (5.5)	45 (6.0)		
Number of hobbies*					0.12	0.94
≥3	380	349 (91.8)	26 (6.9)	5 (1.3)		
2	772	666 (86.3)	74 (9.6)	32 (4.1)		
≤ 1	864	751 (86.9)	65 (7.5)	48 (5.6)		

^*^Refers to Kruskal-Wallis H test; otherwise, it is Mann-Whitney U test; H/Z is the statistical parameter of Kruskal-Wallis H test and Mann-Whitney U test, respectively; The severity of ADL was classified into three levels: normal (14 scores), somewhat impaired (15–21 scores), and severely impaired (≥22 scores).

Table 4 shows the differences in ADL functions of older people with T2DM by family characteristics. We found ADL functions were significantly affected by living conditions (*H* = 28.20, *P* < 0.001), family support (*H* = 19.33, *P* < 0.001), and family income (*H* = 31.51, *P* < 0.001). Moreover, results of trend analysis suggest that more family support (*Z* = –6.07, *P* < 0.001) and better economic status (*Z* = –3.99, *P* < 0.001) were associated with fewer ADL function restrictions.

**Table 4:** Comparison of activities of daily living (ADL) functions by family characteristics in older people with type 2 diabetes in Fuzhou.

**Variable**	***n***	**ADL**			***H***	***P*-value**
		**Normal (*n* = 1,766)**	**Somewhat impaired (*n* = 165)**	**Severely impaired (*n* = 85)**		
Living with*					28.2	< 0.001
Spouse	841	759 (90.3)	60 (7.1)	22 (2.6)		
Children	298	244 (81.9)	29 (9.7)	25 (8.4)		
Spouse and children	746	646 (86.6)	66 (8.8)	34 (4.6)		
Alone	122	112 (91.8)	9 (7.4)	1 (0.8)		
Family support*					19.33	< 0.001
Aged care home	9	5 (55.6)	1 (11.1)	3 (33.3)		
Very	1,208	1,088 (90.1)	91 (7.5)	29 (2.4)		
General	729	609 (83.5)	70 (9.6)	50 (6.9)		
Lack	79	69 (87.3)	4 (5.1)	6 (7.6)		
Family income*					31.51	< 0.001
High	1,259	1,133 (90.0)	93 (7.4)	33 (2.6)		
Average	519	419 (80.7)	60 (11.6)	40 (7.7)		
Low	238	214 (90.0)	12 (5.0)	12 (5.0)		

^*^Refers to Kruskal-Wallis H test; otherwise, it is Mann-Whitney U test; H/Z is the statistical parameter of Kruskal-Wallis H test and Mann-Whitney U test, respectively; The severity of ADL was classified into three levels: normal (14 scores), somewhat impaired (15–21 scores), and severely impaired (≥22 scores).

As shown in Table 5, among the 2016 participants with T2DM, 82.7% of them lived with other chronic diseases. Moreover, the results of trend analysis suggest that those with more existing chronic diseases had more ADL function restrictions (*Z* = 3.38, *P* = 0.001). In addition to T2DM, we found the following two chronic diseases may compromise older people's ADL functions: stroke (*Z* = –3.40, *P* < 0.001) and malignant tumors (*Z* = –2.63, *P* = 0.01).

**Table 5:** Comparison of activities of daily living (ADL) functions by different chronic diseases in older people with type 2 diabetes in Fuzhou.

**Variable**	***n***	**ADL**			***H* /*Z***	***P*-value**
		**Normal (*n* = 1,766)**	**Somewhat impaired (*n* = 165)**	**Severely impaired (*n* = 85)**		
Number of chronic diseases*					5.53	0.06
1	349	314 (90.0)	26 (7.4)	9 (2.6)		
2	1,274	1,119 (87.8)	107 (8.4)	48 (3.8)		
≥3	393	333 (84.7)	32 (8.2)	28 (7.1)		
**Hypertension**					−1.59	0.11
No	444	398 (89.6)	36 (8.1)	10 (2.3)		
Yes	1,572	1,368 (87.0)	129 (8.2)	75 (4.8)		
**Coronary heart disease**					−1.85	0.07
No	1,908	1,677 (87.9)	157 (8.2)	74 (3.9)		
Yes	108	89 (82.4)	8 (7.4)	11 (10.2)		
**Stroke**					−3.4	0
No	1,988	1,747 (87.9)	162 (8.1)	79 (4.0)		
Yes	28	19 (67.9)	3 (10.7)	6 (21.4)		
**Hyperlipidemia**					−0.01	0.99
No	1,879	1,646 (87.6)	154 (8.2)	79 (4.2)		
Yes	137	120 (87.6)	11 (8.0)	6 (4.4)		
**Cerebrovascular disease**					−1.62	0.11
No	1,995	1,750 (87.7)	162 (8.1)	83 (4.2)		
Yes	21	16 (76.2)	3 (14.3)	2 (9.5)		
**Malignant tumor**					−2.63	0.01
No	1,997	1,753 (87.8)	162 (8.1)	82 (4.1)		
Yes	19	13 (68.4)	3 (15.8)	3 (15.8)		
**Cor pulmonale**					−1.06	0.29
No	2007	1,759 (87.6)	165 (8.2)	83 (4.2)		
Yes	9	7 (77.8)	0 (0.0)	2 (22.2)		
**Chronic bronchitis**					−0.86	0.39
No	2000	1,753 (87.6)	164 (8.2)	83 (4.2)		
Yes	16	13 (81.3)	1 (6.2)	2 (12.5)		
**Osteoarthropathy**					−0.84	0.4
No	1922	1,686 (87.7)	158 (8.2)	78 (4.1)		
Yes	94	80 (85.0)	7 (7.5)	7 (7.5)		
**Cataract**					−1.28	0.2
No	1967	1,726 (87.7)	159 (8.1)	82 (4.2)		
Yes	49	40 (81.6)	6 (12.3)	3 (6.1)		
**Glaucoma**					−1.26	0.21
No	2008	1,760 (87.7)	165 (8.2)	83 (4.1)		
Yes	8	6 (75.0)	0 (0.0)	2 (25.0)		
**Chronic gastroenteritis**					−0.02	0.99
No	1968	1,724 (87.6)	161 (8.2)	83 (4.2)		
Yes	48	42 (87.5)	4 (8.3)	2 (4.2)		
**Cholecystitis/Gallstones**					−1.56	0.12
No	1982	1,739 (87.7)	162 (8.2)	81 (4.1)		
Yes	34	27 (79.4)	3 (8.8)	4 (11.8)		
**Emphysema**					−0.25	0.81
No	2009	1,760 (87.6)	165 (8.2)	84 (4.2)		
Yes	7	6 (85.7)	0 (0.0)	1 (14.3)		
**Asthma**					−0.42	0.68
No	2010	1,761 (87.6)	165 (8.2)	84 (4.2)		
Yes	6	5 (83.3)	0v (0.0)	1 (16.7)		
**Fatty liver**					−1.01	0.31
No	1983	1,739 (87.7)	161 (8.1)	83 (4.2)		
Yes	33	27 (81.8)	4 (12.1)	2 (6.1)		
**Other chronic diseases**					−3.37	0
No	1959	1,724 (88.0)	158 (8.1)	77 (3.9)		
Yes	57	42 (73.7)	7 (12.3)	8 (14.0)		

^*^Refers to Kruskal-Wallis H test; otherwise, it is Mann-Whitney U test; H/Z is the statistical parameter of Kruskal-Wallis H test and Mann-Whitney U test, respectively; The severity of ADL was classified into three levels: normal (14 scores), somewhat impaired (15–21 scores), and severely impaired (≥22 scores).

Table 6 summarizes the results of multivariate unconditional logistic regression analysis to identify factors affecting ADL functions. We found the following sub-groups were more likely to suffer from ADL impairment: those aged 70 and over years old (OR = 1.99, 95%CI 1.77–2.56), living in an aged care house or with spouse/children (OR = 2.31, 95%CI 1.25–4.26), low monthly income (OR = 1.49, 95%CI 1.28–1.64), without health insurance (OR = 1.82, 95%CI 1.40–2.40), tight family expenses (OR = 1.95, 95%CI 1.42–2.69), having stroke (OR = 6.70, 95%CI 2.22–20.23) or malignant tumor (OR = 4.45, 95%CI 1.27–15.53), irregular eating habit (OR = 2.55, 95%CI 2.23–2.92), smoking (OR = 1.40, 95%CI 1.22–1.60), sedentary lifestyle (OR = 2.04, 95%CI 1.46–2.85), lack of physical exercise (OR = 1.35, 95%CI 1.19–1.53), sleeping difficulty (OR = 1.25, 95%CI 1.10–1.42), and lack of family support (OR = 1.19, 95%CI 1.10–1.29).

**Table 6:** Identification of factors affecting activities of daily living (ADL) limitation in older people with type 2 diabetes in Fuzhou.

**Variable**	**Reference**	**Comparison group**	***OR* (95%*CI*)**	***P*-value**
Age (years)	60–69	70–79	1.99 (1.37–2.90)	0.00
		≥80	4.78 (3.01–7.60)	0.00
Marital status	Married	Not married	2.31 (1.25–4.26)	0.01
Monthly income (Chinese Yuan)	>2,000	1,000–2,000	1.65 (1.27–2.15)	0.00
		< 1,000	2.66 (2.12–3.32)	0.00
Health insurance	Covered	Partially	1.76 (0.96–3.22)	0.07
		Self-payment	3.15 (1.10–8.98)	0.03
Family expenses	Adequate	Average	1.40 (1.02–1.92)	0.04
		Tight	1.95 (1.42–2.69)	0.00
Stroke	No	Yes	6.70 (2.22–20.23)	0.00
Malignant tumor other diseases	No	Yes	4.45 (1.27–15.53)	0.02
	No	Yes	4.53 (1.90–10.83)	0.00
Eating habit	Very regular	Regular	3.35 (2.37–4.73)	0.00
		Relatively irregular	5.09 (2.90–8.96)	0.00
Smoking	No	Ex-smoker	2.08 (1.13–3.83)	0.02
Sedentary hours		Yes	2.02 (1.16–3.53)	0.01
	< 8	≥8	2.04 (1.46–2.85)	0.00
Doing exercise	No	Occasionally	0.70 (0.46–1.09)	0.11
		Frequently	0.59 (0.37–0.94)	0.03
Sleeping difficulty	No	Occasionally	2.15 (1.49–3.12)	0.00
		Frequently	1.53 (0.85–2.76)	0.16
Living with	Alone	Aged care home	20.20 (3.44–118.40)	0.00
		Spouse	3.26 (1.35–7.84)	0.01
		Children	2.85 (1.26–6.44)	0.01
		Spouse and children	4.09 (1.70–9.83)	0.00

## Discussion

Physical disability is a major socioeconomic and public health issue, as it not only diminishes the quality of life of those affected but also may result in a greater increase in healthcare services utilization such as physician visits and hospitalizations (4, 20). Diabetes is associated with functional disability through mechanisms such as decreased cardiopulmonary reverse, inflammatory or sarcopenic process, extreme of blood glucose, muscle catabolism, cognitive impairment, and inflexible treatment regimens (5, 21–23). Presence of diabetes and associated complications can lead to a significant decline in physical functioning, especially among older patients (8, 24). Currently, there are limited studies in China investigated to what extent older T2DM patients' activities of daily living were affected and its influencing factors. The limitations in ADL and IADL have been widely used as an indicator to assess disability in basic life activities among the population over 65 years old (25, 26). In this study, we found unhealthy lifestyle had significant impacts on older T2DM patients' functional limitations. Specifically, a high level of physical activity, being married, regular eating habits, and non-smoking are protective factors for performing ADLs. On the contrary, a sedentary lifestyle, suffering from stroke or malignant tumor, and sleeping difficulty may increase the risk of ADL limitations among older T2DM patients. We also found that household composition was associated with physical limitations in ADLs. Participants living alone performed ADLs much better than those lived in aged care homes or living with spouse or/and children, probably because those with severe ADL impairment were lack of self-care capability and had to live with others. These findings may provide useful information for the development of nursing practice and the improvement of effective health management for older T2DM patients.

Currently, most published epidemiological research findings support that diabetes was associated with ADL limitations (27). According to a cohort study from China, the risk of ADL impairment was increased by 102% (HR = 2.02, 95%CI 1.29–3.17) for T2DM patients aged 65–74 years, compared to those without T2DM in the same age group (28). Nevertheless, inconsistency still exits. Results of a multi-country study showed that diabetes was not associated with ADL limitations in China after controlling for confounding factors such as socioeconomic status, but significant associations were found in Mexico, Barbados, Brazil, Chile, Cuba, and Uruguay (29).

In this study, we found the prevalence of functional limitations (ADL) among the older T2DM patients in Fuzhou was 12.4%. It is much lower than the national average ADL impairment rate (32.3%) according to the survey data from China Health and Retirement Longitudinal Study (CHARLS) (30, 31). The differences in the prevalence of ADL disability among T2DM patients across studies have been reported by international literature as well (8, 29), which may be due to the varied criteria used to define functional limitations. Moreover, differences in socioeconomic and healthcare services levels (e.g., early diagnosis, medical treatment, and rehabilitation) across regions/cities may also contribute to the disparity. Another possible explanation is that the CHARLS survey data were collected between 2015 and 2016, and evidence has shown that the incidence of ADL disability among the Chinese older adult population with T2DM had a declining trend over time (26), mainly due to the considerable improvements in living standard, biological environment, and healthcare services.

We found ADL limitations increased with age, as older people were more likely to experience T2DM-related comorbidities (32). Moreover, our results showed that more comorbidities were associated with a greater risk of developing ADL limitations. It is in line with previous studies (8, 26). As to gender differences in ADL limitations among T2DM patients, there is no consistency. Most previous literature suggests that older female T2DM patients usually reported more ADL functional limitations and physical disability than their male counterparts (8, 26, 28), although women generally utilized healthcare services more often than men. The greater prevalence and severity of arthritis and musculoskeletal disease among older women may partly explain the difference (33). Another explanation is that women were more likely to report or over-report their ill health and disability than men (34). However, we found males reported more ADL limitations than their female counterparts, probably because males were older than females in this study.

Our results also indicate that those living with a low socioeconomic status were at higher risk of developing functional limitations in ADLs. It is consistent with previous studies (27). Moreover, lower socioeconomic status in older age seems to predict ADL limitations more than socioeconomic status at younger age (27). In recent years, some social security programs have been launched or reformed by the government to provide better welfare to the older population, especially in health. The coverage of basic pension insurance has expanded to about one billion people in 2020 (35). Currently, there are three categories of government-funded health insurance programs, namely urban employee medical insurance, urban resident medical insurance, and new rural cooperation medical insurance, with aims to improve the accessibility for medical treatment. However, social medical insurance schemes in China adopts the “payment-before-reimbursement” principle. The insurers are required to pay the medical expenses in advance when seeking medical treatment, then a certain proportion of medical expenses are reimbursed after treatment. A large amount of prepayment may become one of the reasons restricting low-income groups from seeking timely medical treatment (36), which may potential increase risk of ADL limitations due to lack of heath care access. To reduce the healthcare burden for those with serious chronic diseases, in recent years the reimbursement cap for more than 20 chronic diseases including diabetes has been increased to 140,000 Yuan per year, compared to 6,000 Yuan for general diseases in outpatient clinics (37). Targeted supportive policies for those vulnerable subgroups are helpful for maintaining T2DM patients' ADL functions.

There are several limitations to this study. First, some older T2DM patients with severe functional limitations such as having mobility problems or staying in bed may not go to the community service center during the study period and are possibly under-represented. This may lead to the ADL function impaired rate underestimated. Second, evidence has shown that older patients in poorer health were more likely to participate in health services related research (38). Patients who gave explicit written consent may mischaracterize the health status of the larger population. In this study, we did not count how many people were excluded due to not meeting the inclusion criteria or refusing to participate. It is unclear how people who refused differ from those who agreed to participate. Therefore, the generalization of the results should be cautious due to the potential selection bias. Third, cautious should be exercised if extending the results to rural communities. Lastly, the duration of participants' diseases may be associated with functional limitations in ADLs. However, we did not take it into account in the analysis due to unavailability issue.

## Conclusion

The growing number of older T2DM patients coupled with a rapidly aging population continues to be a major public health concern in China. Older adults with T2DM especially among those aged ≥70 years had a high prevalence of functional limitations across a range of daily living tasks, which not only affect individual life of quality but also present a huge burden on the family, health services system, and the whole society. Identified factors associated with ADL limitations may provide useful information for targeted nursing practice and health promotion.

## Data availability statement

The data collected during the current study is not publicly available as the ethics approval only allows for members of the research team access. Upon reasonable request and with permission of the ethics committee, access can be granted. Any queries should be directed to the corresponding authors.

## Ethics statement

The studies involving human participants were reviewed and approved by Medical Ethics Committee of Fujian Health College. The patients/participants provided their written informed consent to participate in this study.

## Author contributions

J-HJ, JX, L-NJ, and H-LZ conceived the study. J-HJ, DL, and H-LZ designed the questionnaire. J-HJ, DL, L-NJ, YC, YY, and BZ did the field work and collected the data. J-HJ, DL, YC, YY, and BZ entered and cleaned the data. J-HJ, JX, and DL analyzed the data. J-HJ drafted the manuscript. JX, DL, L-NJ, YC, YY, CW, BL, RX, and H-LZ reviewed and edited the manuscript. All authors read and approved the final manuscript.

## Funding

The study was funded by the Medical Innovation Project of Fujian Province (2018-CXB-16) and the Applied Technology Collaborative Innovation Research Project of Fujian Health College (2019-5-1).

## Conflict of interest

The authors declare that the research was conducted in the absence of any commercial or financial relationships that could be construed as a potential conflict of interest.

## Publisher's note

All claims expressed in this article are solely those of the authors and do not necessarily represent those of their affiliated organizations, or those of the publisher, the editors and the reviewers. Any product that may be evaluated in this article, or claim that may be made by its manufacturer, is not guaranteed or endorsed by the publisher.
